# Prevention of retinopathy in type 1 diabetes: a systematic review and meta-analysis

**DOI:** 10.1186/1687-9856-2015-S1-O47

**Published:** 2015-04-28

**Authors:** Sohaib Virk, Kim Donaghue, Tien Wong, Maria Craig

**Affiliations:** 1 The Children’s Hospital at Westmead, Sydney, NSW, Australia; 2 University of New South Wales, Sydney, NSW, Australia; 3 University of Sydney, Sydney, NSW, Australia; 4 Singapore Eye Research Institute, Singapore, Singapore

## 

Diabetic retinopathy (DR) is the most serious ocular complication of type 1 diabetes (T1D) and leading cause of acquired blindness in working aged adults. Although various interventions have been trialled to prevent the development or progression of DR, the evidence to support many of these remains unclear. We systematically reviewed the evidence for primary and secondary interventions, to guide the management of DR in people with T1D.

Systematic searches were performed using MEDLINE, EMBASE and CENTRAL databases (from January 1990 to June 2014) to identify randomised controlled trials and controlled cohort studies reporting the incidence or progression of DR following administration of systemic interventions. English-language studies with a minimum follow-up of one year were eligible. Meta-analyses of extracted data were performed to determine pooled relative risk (RR) reduction.

Twenty-three studies met the inclusion criteria. Intensive insulin therapy significantly reduced the risk of both incident DR (RR 0.44, 95% CI 0.22-0.86, p=0.02) and progression of DR (RR 0.55, 0.31-0.97, p=0.04) compared with conventional therapy. Continuous subcutaneous insulin infusion (CSII) pumps provided significantly greater protection than multiple daily injection therapy (RR 0.33, 95% CI 0.19-0.57, p<0.0001). Angiotensin-converting enzyme inhibition had no impact on DR incidence but reduced progression (RR 0.57, 95% CI 0.34-0.94, p=0.03). Conversely, angiotensin receptor blockade was effective in decreasing DR incidence (RR 0.65, 95% CI 0.49-0.85, p=0.002) but had non-significant effect on progression. Both pancreas-alone and combined pancreas-kidney transplantation retarded progression of DR (RR 0.20, 95% CI 0.10-0.41, p<0.0001). Islet cell transplantation provided no benefit compared with either intensive or conventional insulin therapy.

In people with T1D, there is strong evidence supporting intensive insulin therapy for prevention of DR. Anti-hypertensives also provide protection against DR in normotensive, normoalbuminuric adults but their effectiveness in other populations is yet to be investigated. In patients with T1D of longer duration, pancreas transplantation slows progression of DR. There is insufficient evidence to recommend the use of anti-lipid therapy or other medical interventions.

